# Live-odds gambling advertising and consumer protection

**DOI:** 10.1371/journal.pone.0216876

**Published:** 2019-06-10

**Authors:** Philip W. S. Newall, Ankush Thobhani, Lukasz Walasek, Caroline Meyer

**Affiliations:** 1 Applied Psychology, WMG, University of Warwick, Coventry, United Kingdom; 2 Department of Psychology, University of Warwick, Coventry, United Kingdom; McGill University, CANADA

## Abstract

In-play gambling is a recent innovation allowing gambling to occur during the course of a sporting event, rather than merely before play commences. For years, in-play gambling has been marketed in the UK via adverts displaying current betting odds during breaks in televised soccer, e.g., “England to score in the first 20 minutes, 4-to-1.” Previous research shows that this so-called “live-odds” advertising is skewed toward complex events with high profit margins which consumers do not evaluate rationally. Recent UK regulatory guidance on “impulsiveness and urgency,” aiming to enhance consumer protection around gambling advertising, states that gambling advertising should not “unduly pressure the audience to gamble.” We explored the frequency and content of live-odds advertising over the 2018 soccer World Cup, as a case study of the first major televised sporting event after the publication of this UK regulatory guidance. In total, 69 live-odds adverts were shown over 32 matches (*M* = 2.16 per-match), by five bookmakers. We identified two key features that made advertised bets appear more urgent than necessary. First, 39.1% of bets could be determined before the match ended. Second, 24.6% of bets showed a recent improvement in odds, including a 15.9% subset of “flash odds,” which were limited in both time and quantity. Advertised odds were again skewed toward complex events, with a qualitative trend toward greater complexity than at the previous World Cup. We believe that consumers should be protected against the targeted content of gambling advertising.

## Introduction

Technology and legislation have transformed the UK’s gambling scene in recent years. Soccer gambling used to be relatively low frequency, with bets being made in person or via telephone, and most matches held on Saturday afternoons. Nowadays, bets can be placed either online or on mobile devices, and on international matches around the clock. And with “in-play” gambling, bets can be placed during the course of a sporting event, as odds update in real time with the ebb and flow of play. In this paper we focus on “live-odds” gambling adverts, which show the latest in-play betting odds during breaks in play as a televised sporting event is happening. Live-odds adverts are just one of many gambling advertising techniques. Public concern is mounting over the quantity and content of gambling advertising, which has slowly increased in frequency since its introduction via the Gambling Act 2005. Indeed, 17% of all 2018 soccer World Cup advertising was for gambling [1], and gambling logos can be seen frequently even in the non-commercial BBC’s soccer highlights show [2]. Such widespread advertising makes consumer protection an important issue. One move toward greater consumer protection came from the recent regulatory guidance on “impulsiveness and urgency,” stating that:

“In order not to encourage gambling behaviour that is irresponsible, marketing communications should not unduly pressure the audience to gamble, especially when gambling opportunities offered are subject to a significant time limitation.” [3], p.6.

This guidance was announced in early 2018 before coming into force on April 2^nd^, 2018. Initial reporting speculated that live-odds adverts might consequently be banned [4]. Live-odds adverts are by their very nature limited to the time horizon of the relevant sporting event. However, it is now clear that this guidance only led to minor presentational changes in live-odds adverts. Betting odds used to be accompanied with words to the effect of, “bet now” or, “bet in-play now.” But live-odds adverts continued as before post-guidance, just with the removal of phrases like these [5].

Soccer betting has a traditional baseline bet which should be familiar to many readers [6]. Each soccer match has three main outcomes: either the home team will win, the away team will win, or the match will end in a draw. “Home-draw-away” bets are a set of odds corresponding to the payoffs from successfully betting on each of these three events. Unlike other consumer products such as smartphones or beer brands, there is no key feature distinguishing a home-draw-away bet between different bookmakers, and odds comparison sites allow gamblers to find the bookmaker offering the most attractive odds. Only 7.8% of the live-odds advertising shown by three bookmakers over the previous World Cup in 2014 was for home-draw-away bets [7]. Instead, a majority of live-odds advertising focused on what we call “complex” bets. Complex bets on more specific outcomes can often be created via small changes to the traditional home-draw-away bet. For example, a bet can be advertised on the home team to win by exactly three goals to nil, called a “correct score” bet here, which featured in 35.9% of World Cup 2014 live-odds advertising [7]. Complex bets, such as correct score bets, can naturally offer bigger payoffs on successful bets, which consumers might find attractive. “First/next goalscorer” bets are another complex bet category, requiring bettors to identify the specific player to score the first/next goal out of the 20 outfield players in any one soccer match. First/next goalscorer bets featured in 38.8% of World Cup 2014 live-odds advertising [7]. Overall, live-odds advertising over the previous World Cup steered away from traditional home-draw-away soccer bets.

Live-odds advertising content might be targeted, but would following the bookmakers’ recommendations give gamblers good returns? This question can be answered either by simulating the returns on a past betting strategy, or by inferring returns indirectly via quantifying the inconsistencies in betting odds [8]. Betting odds reveal that the house margin on home-draw-away bets was a constant 10.5% in the late 1990s [9], before falling to a range of 5–6% in the mid-2010s [10,11]. Betting odds from the mid-2010s reveal a much higher house margin in a range of 21.9%–23.2% for correct score bets, and 32.3%–34.6% for first/next goalscorer bets [7,12]. Simulation results using five years of English Premier League data from 2013 onwards reveal similar house margins of 7.1% for home-draw-away bets and 34.3% for correct score bets [13]. By comparison, the house margin in European roulette is 2.7%, which forms the basis of many electronic gambling machine games. Picking the bets featuring the most frequently in live-odds adverts could increase a gamblers’ rate of losses by a multiple of around five times compared to traditional soccer bets, or around 12 times compared to roulette.

Live-odds advertising might be targeted toward high margin products, but are soccer fans aware of the risks? The proper evaluation of product risk is a key principle underlying the theory of responsible gambling [14]. If soccer fans are evaluating risks rationally then some minimal conditions must be satisfied: for example, subjective probabilities must sum to 100%. If there are two possible states of the world, then a rational forecast which puts the probability of rain at 60% must also put the probability of no-rain at 40%. A set of probabilities summing to above 100% is termed “incoherent,” as this can lead to a decision maker accepting a string of bets that are guaranteed to lose money [15]. Across a sequence of studies, a majority of soccer fans were found capable of forming home-draw-away expectations that met this minimal standard of rationality, with sums averaging between 103–112%. However, fans’ forecasts were much worse for correct score events, with sums between 279–306%, and sums of up to 248% for first goalscorer events. Most soccer fans cannot form these minimally-rational evaluations of the complex events dominating live-odds adverts. Arguably, these fans will be poorly informed of the substantial differences in product risk, which could be argued to violate regulatory guidance on, “limitations on the capacity to understand information,” [3], p.6.

Taken together, complex live-odds appear to have both higher levels of objective harm and higher levels of consumer misunderstanding. However, there are other potential misunderstandings that bookmakers might exploit to make high margin products appear better than they really are [16]. Consider one example of a live-odds advert shown immediately before kickoff during the England versus Colombia knockout match, which was seen by 23.8 million viewers [17]:

“England to score in the first 20 minutes. 4-to-1.”

Betting odds of 4-to-1 mean that every £1 staked could win £4 profit if successful [8]. These are much higher than the odds which would have been available on England scoring in the whole match. Many gamblers might have a rough idea of England’s chances of scoring in the match, but it’s a more “complex” calculation to evaluate England’s scoring chances within 20 minutes [18]. England scoring is an easily imaginable “representative” outcome against a weaker team such as Colombia, however, and so many gamblers may just assume that the bet is attractive when presented with such a complex calculation [19,20]. In addition, many gamblers may not think rationally when it comes to betting on their own team, exhibiting an “own-team” bias [21,22]. The odds presented above were subject to time pressure, being valid only if a gambler immediately took out their mobile device and placed a bet via the bookmaker’s app. This (losing) bet was also determined well before the match ended, meaning that gamblers could try to recover their losses via further in-play bets (the match was eventually won by England on penalties after extra time).

In this paper, we evaluate the key features of live-odds gambling advertising shown during the 2018 World Cup. This was the first major televised sporting event after new regulatory guidance aimed to enhance consumer protection in this area was introduced [3]. The phrasing of the guidance is open to interpretation, using qualifiers such as, “not *unduly* pressure the audience to gamble” and, “an *unjustifiable* sense of urgency” [3], p.6. For this reason, we cannot state whether specific adverts strictly complied with or violated the new guidance. Therefore, for the present contribution our aim was to measure and record the content of World Cup 2018 live-odds advertising which seemed relevant to this new guidance and to the previous literature on soccer betting and live-odds advertising, including a previous study of the 2014 World Cup [7].

## Method

One research team member retrospectively viewed all 32 2018 World Cup matches shown on ITV via Box of Broadcasts, and coded the content of broadcasted gambling adverts (The BBC does not show commercial advertising breaks during its programming, meaning that only half [32] of the 2018 World Cup’s 64 matches were analyzed).

Certain aspects of gambling advertising content can change frequently. Therefore, the following high-level categories of live-odds advertising were recorded in the initial round of coding performed by one research team member:

Match. The two national teams who were playing when the live-odds advert was broadcast.

Segment. Whether the live-odds advert was shown pre-match, during the half-time break, or after the 90 minutes of regular play.

Bookmaker. Which bookmaker showed the live-odds advert.

Odds. The odds of the advertised bet, converted into an implied probability [8]. For ease of comparison, these implied probabilities will be inverted in the Results section into the resulting “Decimal odd,” representing the total potential win from a bet of £1. Larger potential wins correspond to lower implied probabilities. Decimal odds are generally considered as a simpler method of communicating odds than the British fractional odds system used in live-odds advertising [8].

Summary. A short textual summary of the advert’s content.

Key offer. A short textual summary of the advertised bet.

After this initial round of coding, a second research team member independently recoded 3 matches (approximately 10% of the sample). The two research team members were in complete agreement on the number and content of live-odds adverts in this sub-sample. The research team then met to discuss the recorded features of live-odds advertising. After this discussion, the following additional categories were added in a secondary round of coding:

Upcoming events. Whether the advert was relevant to the match that was currently being broadcast, or whether the advert was relevant to an upcoming match.

Determined before match end. Whether the bet could become worthless before the end of the match, e.g., “England to score in the first 20 minutes,” or whether the bet’s payoff would be determined at the end of the match. This category was coded conservatively. Some bets could be determined before the match ends if match event makes the bet impossible to payoff (e.g., “Russia to win 3–1,” and the other team scores two goals). This category was restricted to only bets with either definite time limits (e.g., “England to score in the first 20 minutes”), or bets on the *next* event to occur in the match (e.g., “Neymar to score next”).

Type of bet. After the initial data was inputted, we attempted to perform a secondary level of coding where similar bets were clustered together. Any such coding scheme must trade-off the specificity and number of coding categories. We decided on the following categories:

Final scoreline. E.g., “Brazil to win 3–1, 16-to-1.”

Team to score in 90 minutes. E.g., “England to score in 90 minutes, 11-to-10.”

A specific player scoring. E.g., “Ronaldo to score any time tonight, now 5-to-3.”

Penalty shootout. E.g., “Sweden vs. England. A penalty shootout to occur, 6-to-1.”

Complex. Any advertised bet requiring a more specific combination of events to occur. E.g., “Robert Lewandowski and Sadio Mane both to score, 9-to-1.”

Odds changing. Whether the odds were shown as recently changing (any changes were shown as the odds improving, therefore implying a large payoff if the specified event were to happen).

Flash odds. Whether the recently improved odds were described as “flash odds.” Further description of how flash odds work was found on the bookmaker William Hill’s website in August 2018, describing how flash odds are limited both in time and based on their popularity:

“Flash Odds are hugely enhanced prices available for a limited time, which means that if you’re not quick enough, they could be gone in a flash.”

“They offer a sudden opportunity to take advantage of a sizeably-enhanced price on a popular market, but the amount of bets William Hill will take at these generously-inflated fractions can only ever be finite. … Flash Odds are prices that are available on popular markets and events for a limited time only. They can appear when you least expect them to.”

Since an earlier version of this paper was posted online as a preprint, which is accessible from https://psyarxiv.com/3uc9s/, a second dataset coded by a Guardian journalist was made available to us [1]. This second dataset covers the first 30 matches in the original data, and covers the advertising breaks shown from just before, until just after the end of the match. By comparison, the coding presented in this paper is more inclusive, covering all of the advertising breaks shown on the Box of Broadcasts transmission. Comparing the two datasets led to an increase of six live-odds adverts, for an inter-rater agreement rate of 90.5%, above the suggested 70% threshold for percentage agreement [23]. The data presented in this paper can be found at https://osf.io/xnkgq/. The practice of pre-publication peer-review via preprints is becoming increasingly popular [24], and we believe that this paper was improved via this process.

## Results

In total, 69 live-odds adverts (*M* = 2.16 per-match) were shown by five bookmakers, which are summarized in Table 1. A majority of adverts were shown during the half-time break (53.6%), 22 adverts were shown before a match started (31.9%), and 10 adverts were shown after a match finished (14.5%, and therefore related to an upcoming match). The average decimal odds were 7.4, meaning that a successful bet of £1 would on average win £7.40 in total [8]; Bet 365 was the bookmaker with the highest average odds, of 9.8.

**Table 1:** Content analysis summary.

Feature		Bet365	Betfair	Coral	Ladbrokes	William Hill	Total
Timing	Pre-	11	0	1	2	8	22
	Half-time	17	2	3	1	14	37
	Post-	3	2	0	0	5	10
Average odds		9.8	6.7	6.5	4.4	6.3	7.4
N determined before match end		18	0	1	1	7	27
Type	Final scoreline	13	0	0	0	0	13
	Team to score in 90 minutes	0	0	0	0	2	2
	A specific player scoring	18	4	0	1	4	27
	Penalty shootout	0	0	2	1	0	3
	Complex	0	0	2	1	21	24
Odds shown as recently improving		0	0	4	2	11	17
“Flash odds”		0	0	0	0	11	11
Total		31	4	4	3	27	69

*Note*: Some live-odds adverts were shown after a match had ended, “post-match,” and these corresponded to an upcoming match. A further nine of the adverts shown pre-match or at half-time corresponded to events relevant to upcoming matches, rather than the match that was currently happening. The first four types of bets, from “Final scoreline” to “Penalty shootout” correspond to bets requiring only the specified event to happen. “A specific player scoring” corresponds to bets involving a specific player scoring either one goal, the next goal, or more than one goal, but with no other conditions required for the bet to payoff. A unique category was created for the most complex bets, as these could require multiple events to happen (e.g., a specific player scoring and a team to win by a specific scoreline).

In total, 27 advertised bets (39.1%) could be determined before the match’s end. For example, the bet described in the introduction was shown by Ladbrokes immediately before kick-off for Colombia versus England, “England to score in the first 20 minutes, 4-to-1,” a match seen by 23.8 million viewers [17]. Coral advertised a bet for both teams to score in the first half, and William Hill advertised 7 bets with this feature, e.g., “Mohamed Salah to score next and over 2 cards in the second half, 10-to-1.” Bet365 advertised 18 bets with this feature; all of these bets were on the identity of the first/next goalscorer, e.g., “Sterling to score the first goal, 11-to-1.” All but one of Bet365’s first/next goalscorer bets were shown at half-time.

In total, 17 advertised bets (24.1%) were shown as having recently improving odds. All of Coral’s four advertised bets had this feature, e.g., “Sweden vs. England, penalty shootout, was 9-to-2, now 6-to-1,” and two of Ladbrokes’s three adverts did, e.g., “Harry Kane to score in the 2nd half, was 13-to-8, now 9-to-4.” William Hill showed 11 odds as recently improving, e.g., “Lionel Messi to score and Argentina to win, was 3-to-1 now 4-to-1.” Furthermore, William Hill’s odds were described as “flash odds”—see a full description of flash odds in the Method section—which meant that these improved odds were limited in both time and the total amount bet by gamblers.

Bets on a specific player to score were the most frequently advertised type of bet (39.1%). Bet 365 was the only bookmaker advertising odds on the final scoreline (18.8%), e.g., “Germany to win 4–0, 25-to-1.” “Complex” bets were the last frequently advertised type of bet (34.8%), and all but three of these adverts were shown by William Hill, e.g., “Brazil to win, Neymar to score, both teams to score, and Xhaka to be carded, 18-to-1.” Several of William Hill’s complex odds also played on own-team bias. For example, “England to win by three or more goals, Harry Kane to score, and over 11 corners, 16-to-1.”

## Discussion

For the present contribution our aim was to measure and record the content of World Cup 2018 live-odds advertising which seemed relevant to the new guidance around “impulsiveness and urgency [3], and to the previous literature on soccer betting and live-odds advertising. The phrasing of the guidance is open to interpretation, using qualifiers such as, “not unduly pressure the audience to gamble” and, “an unjustifiable sense of urgency” [3], p.6. For this reason, we can only describe features of advertised bets, and are unable to state whether specific adverts strictly complied with or violated the new guidance.

We identified two recurring features which seem particularly relevant to recent regulatory guidance on “impulsiveness and urgency” [3]. Some 39.1% of advertised odds could be determined before the end of the match, potentially encouraging repeated in-play betting. Additionally, 24.6% of odds were shown as recently improving, including a subset of “flash odds,” which were limited in both time and quantity. Neither of these features are necessary for a live-odds advert to exist, with for example an advert for a traditional bet on, “England to win” displaying neither feature. Other stakeholders should decide whether these features, when seen in aggregate, constitute an “*unjustifiable* sense of urgency” [3], p.6.

Some features of World Cup 2018 live-odds advertising were similar to the previous World Cup in 2014. As might be evident to soccer fans from the quoted example bets given in the Results section, there was a tendency for “representative” highly-skilled and well-known players and teams to feature in advertised bets. This same pattern of advertised events being representative was also found in 2014 [7]. In total, 58% of advertising was for correct score or specific goalscorer bets (compared to 74.7%; [7]). These are bets with high house margins which soccer fans struggle to form minimally-rational expectations of [12]. By comparison, home-draw-away bets, which have lower house margins and which soccer fans do seem to at least minimally-understand, did not feature at all in 2018 World Cup advertising, after appearing in 7.8% of World Cup 2014 advertising [7]. Only 4% of World Cup 2014 live-odds advertising featured particularly complex bets, e.g. “Thomas Müller to score first and Germany to win 3–1.” By comparison, 34.8% of World Cup 2018 advertising was for adverts of similar levels of complexity. Soccer bets could be categorized in different ways, and we do not believe that these comparisons should be subjected to formal quantitative tests. But there did seem to be a qualitative increase in the complexity of gambles featuring in live-odds advertising since the previous World Cup in 2014.

The present research was limited to being an observational study of gambling advertising content. The present research could not determine how this targeted content might affect gamblers’ behavior. Internationally, there is more evidence on gambling advertising content and perceptions of gambling advertising, than there is evidence on gambling advertising’s effects on behavior [25]. Some Australian evidence suggests that gambling advertising can increase self-reported increases in bet size and frequency [26]. However, these results have not yet been replicated in the UK. The present research is also limited to TV gambling advertising. However, recent figures reveal that now 80% of all UK gambling marketing spending occurs online [27]. Online advertising is increasingly targeted at individuals [28], meaning that researchers simply cannot track the frequency, content, and effectiveness of online gambling advertising as they can with TV gambling advertising. Data on online gambling advertising targeting, content, and frequency exist, and is held by gambling companies and the media platforms that they advertise on. These data should be shared more broadly [29], as one way of effectively studying gambling marketing strategies online.

Gambling is considered a public health issue by many researchers [30–34]. Here we want to provide some observations relevant to live-odds advertising and a public health perspective on gambling. In-play soccer betting appears particularly attractive to problem gamblers [35]. Gambling advertising is subject to a 9PM watershed outside of live sport, making live sport a unique concern for youth gambling [1]. In a 2018 survey, 14% of British 11–16 year-olds had gambled in the previous week, and 66% had seen gambling advertising on TV [36]. Australian research shows how children are influenced by sports gambling advertising [37–39]. On December 6^th^ 2018 it was announced that the British bookmaking industry would voluntarily agree to a pre-watershed “whistle-to-whistle” ban on gambling advertising around live sport, with an exemption for horse racing [40]. If these proposals are enacted, then the patterns observed in this paper should help inform studies of online gambling advertising, which looks set to continue unchecked.

It is interesting to compare responses across different public health crises. In the UK, calorie labelling and alcohol unit labelling are part of the response to obesity and overdrinking. The UK gambling industry has voluntarily included responsible gambling messages as a part of its advertising for some time [41]. However, at present these messages mainly contains the words, “when the fun stops, stop” in bold colors. Consumers are given no numerical information to compare the risks of different soccer bets, akin to calorie or alcohol unit labelling. By comparison, UK electronic gambling machines must disclose the house margin as the return-to-player = (100 –house margin) %. [42]. At a very minimum, similar health warning labels for soccer would reveal that the bets dominating advertising have far higher house margins than traditional soccer bets, and that some soccer bets are more than fifty times worse than other bets [13]. We do not believe this will solve all of the public health issues arising from gambling and soccer, as consumers struggle to understand complex probabilities [19], and this misunderstanding makes it difficult to debias consumers via warning labels [43]. But we view such a step as a minimum requirement if the present industry discourse around consumer protection and responsible gambling is to be seen as more than mere empty rhetoric [44].

## References

[1] Duncan P, Davies R, Sweney M. Children ‘bombarded’ with betting adverts during World Cup. 2018; https://www.theguardian.com/media/2018/jul/15/children-bombarded-with-betting-adverts-during-world-cup, 2018.

[2] Cassidy R, Ovenden N. Frequency, duration and medium of advertisements for gambling and other risky products in commercial and public service broadcasts of English Premier League football. 2017; https://osf.io/gprkv/?action=download, 2017.

[3] Committee of Advertising Practice. Regulatory statement: gambling advertising guidance. 2018; https://www.asa.org.uk/uploads/assets/uploaded/5e19ecb5-a0b2-4322-aeb13047bb408298.pdf.

[4] Keay L. Ray Winstone’s ‘Bet In-Play Now’ TV ads face being banned in problem gambling crackdown. 2018; https://www.dailymail.co.uk/news/article-5392421/Ray-Winstones-Bet-Play-adverts-face-ban.html, 2018.

[5] Ellson A. Watchdog bans Ray Winstone ‘bet now’ adverts during live matches. 2018; https://www.thetimes.co.uk/article/watchdog-bans-bet-now-adverts-during-live-matches-6zbf8wndw, 2018.

[6] Forrest D. Soccer betting in Britain In: Hausch DB, Ziemba WT, editors. Handbook of Sports and Lottery Markets: Elsevier; 2008 p. 421–446.

[7] Newall PWS. How bookies make your money. Judgment and Decision Making 2015;10[3]:225–231.

[8] Cortis D. Expected values and variances in bookmaker payouts: A theoretical approach towards setting limits on odds. The Journal of Prediction Markets 2015;9[1]:1–14.

[9] Kuypers T. Information and efficiency: an empirical study of a fixed odds betting market. Appl Econ 2000;32[11]:1353–1363.

[10] Buhagiar R, Cortis D, Newall PWS. Why do some soccer bettors lose more money than others? Journal of Behavioral and Experimental Finance 2018;18[2018]:85–93.

[11] Constantinou AC, Fenton NE. Profiting from arbitrage and odds biases of the European football gambling market. The Journal of Gambling Business and Economics 2013;7[2]:41–70.

[12] Newall PWS. Behavioral complexity of British gambling advertising. Addiction Research & Theory 2017 2/05;25[6]:505–511.

[13] Hassanniakalager A, Newall PWS. A machine learning perspective on responsible gambling. 2018; 10.31234/osf.io/sxbaq.

[14] Parke A, Harris A, Parke J, Rigbye J, Blaszczynski A. Responsible marketing and advertising in gambling: a critical review. The Journal of Gambling Business and Economics 2015;8[3]:21–35.

[15] Seidenfeld T. Calibration, coherence, and scoring rules. Philosophy of Science 1985;52[2]:274–294.

[16] Newall PWS. Dark nudges in gambling. Addiction Research & Theory 2018.

[17] Ruby J. England viewing figures: World Cup win over Colombia was watched by a staggering 23.8 million on ITV. 2018; https://www.standard.co.uk/stayingin/tvfilm/england-viewing-figures-world-cup-win-over-colombia-was-watched-by-a-staggering-238-million-on-itv-a3878846.html, 2018.

[18] Tversky A, Koehler DJ. Support theory: a nonextensional representation of subjective probability. Psychol Rev 1994;101[4]:547–567.

[19] Tversky A, Kahneman D. Extensional versus intuitive reasoning: The conjunction fallacy in probability judgment. Psychol Rev 1983;90[4]:293–315. 10.3389/fpsyg.2015.01832 PMC4658440 26635712

[20] Tversky A, Kahneman D. Judgment under uncertainty: Heuristics and biases. Science 1974 9 27;185[4157]:1124–1131. 10.1126/science.185.4157.1124 17835457

[21] Massey C, Simmons JP, Armor DA. Hope over experience: Desirability and the persistence of optimism. Psychological Science 2011;22[2]:274–281. 10.1177/0956797610396223 21228135

[22] Morewedge CK, Tang S, Larrick RP. Betting your favorite to win: Costly reluctance to hedge desired outcomes. Management Science 2016;64[3]:997–1014.

[23] Stemler SE, Tsai J. Best practices in interrater reliability: Three common approaches In: Osborne J, editor. Best Practices in Quantitative Methods CA, USA: Sage Publications; 2008 p. 29–49.

[24] Munafò MR, Nosek BA, Bishop DV, Button KS, Chambers CD, du Sert NP, et al A manifesto for reproducible science. Nature Human Behaviour 2017;1[1]. 10.1038/s41562-016-0021 PMC7610724 33954258

[25] Newall PWS, Moodie C, Reith G, Stead M, Critchlow N, Morgan A, et al. Gambling marketing from 2014 to 2018: A literature review. Current Addiction Reports 2019.

[26] Hing N, Russell AM, Thomas A, Jenkinson R. Wagering Advertisements and Inducements: Exposure and Perceived Influence on Betting Behaviour. Journal of gambling studies 2019. 10.1007/s10899-018-09823-y 30604033

[27] GambleAware. Gambling companies spend £1.2 billion marketing online, five times more than on television ads. 2018; https://about.gambleaware.org/media/1857/2018-11-24-gambling-marketing-online-five-times-tv-ad-spend.pdf, 2018.

[28] Matz SC, Kosinski M, Nave G, Stillwell DJ. Psychological targeting as an effective approach to digital mass persuasion. Proc Natl Acad Sci U S A 2017 11 28;114[48]:12714–12719. 10.1073/pnas.1710966114 29133409 PMC5715760

[29] Cassidy R, Loussouarn C, Pisac A. Fair Game: Producing gambling research—The Goldsmiths report. London: Goldsmiths, University of London; 2013.

[30] Markham F, Young M. “Big gambling”: the rise of the global industry-state gambling complex. Addiction Research & Theory 2015;23[1]:1–4.

[31] Orford J. An unsafe bet?: The dangerous rise of gambling and the debate we should be having. Singapore: John Wiley & Sons; 2010.

[32] Adams PJ. Gambling, freedom and democracy. New York: Routledge; 2008.

[33] Livingstone C, Adams PJ. Harm promotion: observations on the symbiosis between government and private industries in Australasia for the development of highly accessible gambling markets. Addiction 2011;106[1]:3–8. 21188851 10.1111/j.1360-0443.2010.03137.x

[34] Lopez-Gonzalez H, Estévez A, Griffiths M. Marketing and advertising online sports betting: a problem gambling perspective. J Sport Soc Iss 2017;41[3]:256–272.

[35] Lopez-Gonzalez H, Griffiths M, Estévez A. In-play betting, gambling severity, and other risks: a survey study of Spanish sports bettors. Communication and Sport 2018.

[36] Gambling Commission. Young People & Gambling 2018: A research study among 11–16 year olds in Great Britain. 2018; https://www.gamblingcommission.gov.uk/PDF/survey-data/Young-People-and-Gambling-2018-Report.pdf, 2018.

[37] Pitt H, Thomas SL, Bestman A, Daube M, Derevensky J. What do children observe and learn from televised sports betting advertisements? A qualitative study among Australian children. Aust N Z J Public Health 2017;41[6]:604–610. 10.1111/1753-6405.12728 29044821

[38] Pitt H, Thomas SL, Bestman A, Daube M, Derevensky J. Factors that influence children’s gambling attitudes and consumption intentions: lessons for gambling harm prevention research, policies and advocacy strategies. Harm Reduction Journal 2017 2/17;14[11]. 10.1186/s12954-017-0136-3 PMC5316223 28212685

[39] Pitt H, Thomas SL, Bestman A, Stoneham M, Daube M. “It’s just everywhere!” Children and parents discuss the marketing of sports wagering in Australia. Aust N Z J Public Health 2016;40[5]:480–486. 10.1111/1753-6405.12564 27524502

[40] Kelner M. Gambling firms back ban on betting adverts during live TV sport. 2018;. Accessed https://news.sky.com/story/ban-on-in-play-tv-gambling-adverts-is-denied-as-betting-stocks-lose-11573019, 2018.

[41] Senet Group. Gambling industry responds to public concerns. 2014; http://senetgroup.org.uk/gambling-industry-responds-to-public-concerns/, 2014.

[42] Parke J, Parke A, Blaszczynski A. Key issues in product-based harmm minimisation: examining theory, evidence and policy issues relevant in Great Britain. London: Responsible Gambling Trust; 2016.

[43] Weiss-Cohen L, Konstantinidis E, Speekenbrink M, Harvey N. Task complexity moderates the influence of descriptions in decisions from experience. Cognition 2018;170:209–227. 10.1016/j.cognition.2017.10.005 29078094

[44] Reith G. Reflections on responsibility. Journal of Gambling Issues 2008[22]:149–155.

